# The Development of Electroconvection at the Surface of a Heterogeneous Cation-Exchange Membrane Modified with Perfluorosulfonic Acid Polymer Film Containing Titanium Oxide

**DOI:** 10.3390/membranes10060125

**Published:** 2020-06-17

**Authors:** Violetta Gil, Mikhail Porozhnyy, Olesya Rybalkina, Dmitrii Butylskii, Natalia Pismenskaya

**Affiliations:** Department of Physical Chemistry, Kuban State University, 149 Stavropolskaya st., 350040 Krasnodar, Russia; porozhnyj@mail.ru (M.P.); olesia93rus@mail.ru (O.R.); dmitrybutylsky@mail.ru (D.B.); n_pismen@mail.ru (N.P.)

**Keywords:** fouling, ion-exchange membrane, surface modification, electroconvection, voltammetry, chronopotentiometry, impedance spectroscopy

## Abstract

One way to enhance mass transfer and reduce fouling in wastewater electrodialysis is stimulation of electroconvective mixing of the solution adjoining membranes by modifying their surfaces. Several samples were prepared by casting the perfluorosulfonic acid (PFSA) polymer film doped with TiO_2_ nanoparticles onto the surface of the heterogeneous cation-exchange membrane MK-40. It is found that changes in surface characteristics conditioned by such modification lead to an increase in the limiting current density due to the stimulation of electroconvection, which develops according to the mechanism of electroosmosis of the first kind. The greatest increase in the current compared to the pristine membrane can be obtained by modification with the film being 20 μm thick and containing 3 wt% of TiO_2_. The sample containing 6 wt% of TiO_2_ provides higher mass transfer in overlimiting current modes due to the development of nonequilibrium electroconvection. A 1.5-fold increase in the thickness of the modifying film reduces the positive effect of introducing TiO_2_ nanoparticles due to (1) partial shielding of the nanoparticles on the surface of the modified membrane; (2) a decrease in the tangential component of the electric force, which affects the development of electroconvection.

## 1. Introduction

The broader use of electro-membrane technologies for separation, concentration, and isolation of valuable substances from wastewater and liquid media of the food industry is constrained by fouling which is an accumulation of mineral and organic pollutants on the surface and in the volume of ion-exchange membranes [1,2,3,4]. Fouling leads to an undesired decrease in useful mass transfer and increase in energy consumption [5]. It reduces the lifespan of membranes and requires spending on their regular cleaning [6]. The result is a significant increase in the cost of the final product. One way to prevent fouling is to stimulate electroconvective mixing of the solution [7,8,9] by targeted selection of ion-exchange membranes, modifying their surface [9] and/or using pulsed current modes [7,8,9].

Nowadays, due to the works of Dukhin, Mishchuk [10,11], Rubinstein, Zaltzman [12,13], Nikonenko, Urtenov [14,15], and a number of other researchers [16,17,18] there are certain concepts of the mechanisms of electroconvection (EC) and those factors that can induce electroconvective vortices. The details of these ideas are described in the review [19]. There are two main mechanisms for the development of EC. Bulk EC-I is due to the effect of an electric field on the residual space charge in a quasi-electroneutral electrolyte solution with an uneven concentration distribution. EC-II is caused by the electroosmotic slip of the space charge region (SCR). This SCR is formed in a depleted solution at the boundary with the membrane. A high degree of hydrophobicity of the membrane surface facilitates the fluid slip and stimulates EC-II [20,21,22].

Both EC-I and EC-II can be of two kinds currently known. (Quasi) equilibrium EC develops at low currents/voltages (*i* ≤ *i*_lim_; *i*_lim_ is the limiting current). In this case, the electrical double layer (EDL) at the membrane/depleted solution boundary is (quasi) equilibrium. The structure of the diffuse part of the EDL retains the Boltzmann distribution of ions which is shifted by an imposed external field [23]. In the case of EC-I, this kind of electroconvection is denoted by the term “electroosmosis of the first kind” [10,11]. Equilibrium EC can develop only at the electrically inhomogeneous and/or curved surface, which stimulates an appearance of the tangential component of an electric force. Its development is facilitated by a high surface charge [24]. In this case, small stable electroconvection vortices arise in the solution adjacent to a membrane. These vortices deliver a more concentrated solution to the depleted diffusion layer. This results in the “negative” conductance of the membrane system, when its resistance near the limiting current becomes lower than at *i* << *i*_lim_, and also in the growth of the experimentally determined limiting current compared to that calculated by the convective-diffusion model [24,25].

In overlimiting current modes (*i* ≥ *i*_lim_), a nonequilibrium EC [13], also called “electroosmosis of the second kind”, develops [11]. An indispensable condition for the development of a nonequilibrium EC is the presence of an extended SCR (which is outside the quasi-equilibrium EDL) and fluctuations in the concentration, electric fields, or the fluid velocity field, which, as a rule, occur in intensive current modes. The reason for the hydrodynamic instability after reaching a certain threshold value of the potential drop is the appearance of a positive feedback between the fluctuation of the local tangential force and the liquid electroosmotic slip velocity [12,26]. As a result, electroconvection vortices become larger than those in the case of equilibrium EC [27]. Their instability with time is the reason for the noticeable oscillations of the current (potential drops) observed in intensive current modes on the current-voltage characteristics (CVCs) and chronopotentiograms (ChPs) [9,28]. Partial removal of the diffusion limitations of salt delivery to the receiving side of the membrane is expressed in a reduction in length and an increase in the slope of the CVC plateau [9,28,29].

Thus, the development of EC is facilitated by electrical [9,28,29,30] and geometric [31,32] heterogeneity of the surface, a high degree of its hydrophobicity [24,25], and also a high electric charge [24]. The specified surface characteristics are achieved by profiling the surface of the membranes [31,32], varying the dispersion of the ion-exchange resin particles in heterogeneous membranes [33,34], and by modifying the membrane surface with various polyelectrolytes [29,30,35], in particular, conductive perfluorosulfonic acid (PFSA) polymer films [9,20,24,28]. Doping such a film with carbon nanotubes enhances electroconvection due to an increase in hydrophobicity and an increase in surface roughness [20]. It is very promising to increase the surface charge by doping the PFSA film with nanoparticles of metal oxides (such as TiO_2_, ZrO_2_, SiO_2_). Currently, these dopants are mainly introduced into the pores of membranes and are widely used to increase their conductivity at low relative humidity in the fuel cells [36,37,38,39] and improving membrane permselectivity [40,41]. Recent studies revealed that the deposition of TiO_2_-doped films on the surface increases the fouling resistance of the ultrafiltration, osmosis, and ion-exchange membranes [42,43,44]. For example, the authors of [44] modified the surface of the anion-exchange membrane by poly (sodium 4-styrene sulfonate) (PSS) with titanium dioxide nanoparticles. They found an increase in the surface charge and its hydrophilicity, as well as the self-cleaning ability of this surface due to photocatalytic decomposition caused by ultraviolet irradiation. Apparently, the effect of such surface modification on the development of electroconvection has not yet been investigated.

The aim of this work is to study the development of electroconvection as a function of the titanium oxide mass fraction in a perfluorosulfonic acid polymer film that modifies the surface of a heterogeneous cation-exchange membrane.

We hope that in the future, such a modification method can be used to create a new generation of ion-exchange membranes that are resistant to sedimentation and fouling during electrodialysis processing of liquid media of the food industry and natural waters.

## 2. Materials and Methods

### 2.1. Membranes

Commercial heterogeneous cation-exchange MK-40 membrane (Shchekinoazot, Pervomayskiy, Tula Region, Russia) is used as the substrate for preparing the modified membrane samples. The MK-40 membrane is produced by hot pressing of a mixture of polyethylene (inert binder) and powdered KU-2 ion-exchange resin (styrene-divinylbenzene sulfonated copolymer) and contain fixed sulfonic acid functional groups. Most of the surface (more than 80%) of the MK-40 membrane is non-conductive [45]. Grains of KU-2 ion-exchange resin, which form crests on the polyethylene surface, provide selective conductivity of the membrane.

The modified membranes were prepared by forming a thin homogeneous film on the MK-40 membrane surface by casting an LF-4SK solution (Plastpolymer, Saint Petersburg, Russia), containing a certain amount of titanium oxide particles. The LF-4SK is a solution of tetrafluoroethylene copolymer with sulfonated perfluorovinylether in isopropyl alcohol. Solvent evaporation gives a film identical to the MF-4SK membrane, a Russian analogue of the Nafion membrane. To prepare the modifier, the required amount of titanium butoxide (EKOS-1, Moscow, Russia) was added to the LF-4SK solution, then the modifier was placed in an ultrasonic bath PSB-2835-05 (Ultrasonic Equipment Center PSB-Gals, Moscow, Russia) to disperse the formed particles.

Modification of the membranes was carried out according to a method similar to that described in [46]. Before applying the modifier, the surface of the MK-40 substrate-membrane was roughened using a fine-grained abrasive sandpaper (P600). Application of the modifier solution on the membrane surface was carried out via drop casting. Preparing the MK-40_21_ sample, an LF-4SK solution containing no titanium oxide particles was used. The MK-40_21_ membrane was used as a reference sample to detect the effect of titanium oxide particles. The characteristics of the commercial and modified membranes are given in Table 1.

### 2.2. Methods

#### 2.2.1. Voltammetry, Chronopotentiometry, and Impedance Spectroscopy

**Figure 1:**
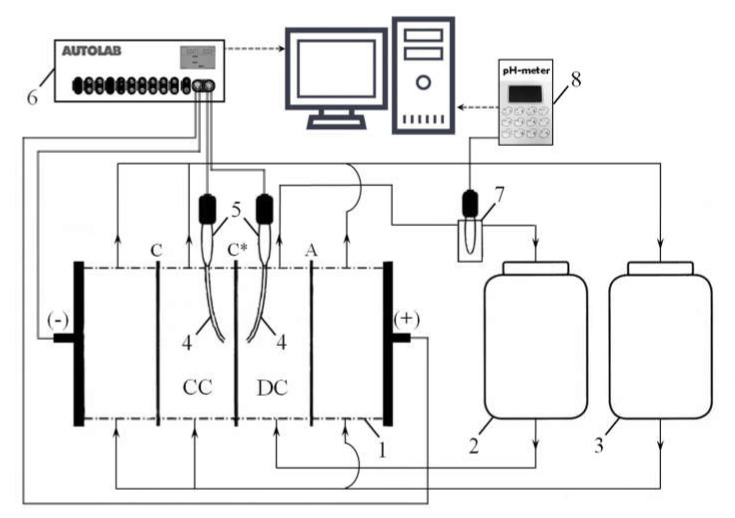
Principal scheme of the experimental setup: electrodialysis cell (1) consisting of one desalination compartment (DC), one concentration compartment (CC) and two electrode compartments; tanks with solutions (2, 3); Luggin’s capillaries (4), connected with silver chloride electrodes (5); electrochemical complex Autolab PGSTAT-100 (6); flow pass cell with pH combination electrode (7), connected to pH-meter Expert 001 (8).

The electrochemical characteristics, the current-voltage curves, chronopotentiograms, and impedance spectra of the studied membranes were obtained using the experimental setup shown in Figure 1, its detailed description is presented in Supplementary Materials. The setup includes a laboratory four-compartment flow electrodialysis cell, described in detail in [45]. The compartments of the cell were formed by the studied cation-exchange membrane (C*) and auxiliary anion-exchange MA-41 (A) and cation-exchange MK-40 (C) membranes. Current was supplied to the cell and the potential drop was measured using an electrochemical complex Autolab PGSTAT-100 (EcoChemi, Utrecht, The Netherlands). The experimental procedure for obtaining ChP and CVC is detailed in [45].

To measure the electrochemical impedance spectra, the closed silver chloride measuring electrodes (immersed in the saturated KCl solution) used in the ChP and CVC measurements were replaced by open silver chloride electrodes (immersed in 0.02 M NaCl solution). It takes about 2 h to get one spectrum. Before recording the complex impedance, the membrane was preliminarily held for 40 min at a given direct current density. Then alternating current was applied. The time required to achieve a quasistationary state at a given frequency was determined automatically by the Autolab PGSTAT-100 complex. Impedance spectrum recording starts at a low frequency equal to 0.003 Hz and ends at a frequency equal to 5 × 10^5^ Hz. From the values of the real (Re*Z*) and imaginary (Im*Z*) components of the impedance of the membrane system obtained at a given frequency and at a given direct current density, the corresponding values (Re*Z* and Im*Z*) obtained at zero direct current (*i* = 0 A) were subtracted. This data processing allows one to exclude from consideration (1) the contribution of the measuring electrode system impedance to the studied membrane system impedance, (2) the ohmic resistance of the membrane and the response of the membrane system to the alternating current bias (the amplitude of alternating current is 0.25 mA). In preliminary experiments, it was shown that in the studied direct current range, polarization phenomena do not significantly affect the impedance of the measuring electrodes. The advantage of such processing of the impedance spectra is the reduction of errors, which occur as the result of electrochemical cell reassembling to determine the impedance of the measuring electrode system.

As a rule, the high-frequency arc [48,49] (in the range from 10^3^ to 1.3 × 10^5^ Hz) dominates the impedance spectrum obtained at zero direct current and presented in Nyquist plot. It characterizes the membrane and interphase boundaries [50,51]. The intercept by this arc on the Re*Z* axis corresponds to the ohmic resistance of the membranes R^Ω^ [48,49,52,53], and the frequency at the maximum of the Im*Z*, *f*_max_, allows one to estimate the effective electric capacitance *C* of the membrane/solution interface by the formula [50]:

(1) C=12πfmax(−ImZmax)

where ‒Im*Z*_max_ is the maximum value of the imaginary impedance component at the high-frequency arc. This capacitance includes the capacitances of the EDLs on the membrane/solution boundaries, as well as the capacity caused by the asymmetry of the depleted and enriched diffusion boundary layers DBLs, which occurs when a direct electric current flows.

If the applied direct current is greater than zero, taking into account the experimental data processing mentioned above, the intercept by the high-frequency arc on the Re*Z* axis gives the ohmic resistance of the diffusion layers. The appearance of the Gerischer arc in the medium-frequency range of the spectrum (10–10^3^ Hz) indicates the presence of an intense chemical reaction at the membrane/solution interface, namely, the generation of H^+^ and OH^–^ ions, catalyzed by fixed groups of membranes [54]. The low-frequency (0.003–10 Hz) arc of the spectrum (finite-length Warburg impedance) characterizes the diffusion and electroconvective transfer of ions in DBL adjacent to a membrane. The DBL thickness can be found from difference in values of real component of the low-frequency arc for the lowest and the highest frequencies [55]. The ratio of the lengths of the intercepts by each of these arcs on the Re*Z* axis gives information on the contribution of each of the phenomena (the formation of concentration profiles near the membrane surface; the appearance and transport of new charge carriers at the membrane/solution interface; a decrease in the diffusion layer thickness due to electroconvection) to electrochemical characteristics of the studied membrane systems [35].

The experiments are conducted at a constant temperature of 25 ± 1 °C.

#### 2.2.2. Diffusion Permeability and Conductivity Measurements

The concentration dependence of membrane conductivity is measured by the differential method using a clip-type cell [56,57] and a MOTECH MT4080 immittance meter at an AC frequency of 1 kHz. The measurements were carried out in NaCl solutions with different concentrations (0.1, 0.25, 0.5, 0.75, 1 M). 

The experimental investigation of the concentration dependence of diffusion permeability was conducted at the same NaCl concentrations. The measurements were carried out using two-compartment cell and conductivity meter Expert 002 (Econix-Expert, Ltd., Moscow, Russia) [58].

The thickness of studied membranes was measured using a digital micrometer Schut Filetta (Schut Geometrical Metrology, Groningen, The Netherlands).

#### 2.2.3. Contact Angles Measurements

The contact angles (*θ*) of the membranes under study were measured using the sessile drop technique [20] described in detail in Supplementary Materials. The studied membrane was in a swollen state, it was removed from the 0.02 M NaCl solution immediately before the measurements. The contact angles reported in this paper were registered 20 s after the applying a drop of distilled water on the membrane surface. 

#### 2.2.4. Surface Roughness Measurements

The surface roughness of the studied membranes was determined by using an optical microscope SOPTOP CX40M (Ningbo Sunny Instruments Co., Ltd., Yuyao, China). The surface of a membrane is shaped of so-called “hills” and “valleys”, corresponding to the highest and lowest areas of the membrane relief. The difference between these two extreme forms of the membrane surface shape, parameter *b* (μm), characterizes the roughness and is measured by moving the objective and focusing on them, the focal length of the system being the same.

## 3. Results and Discussion

### 3.1. Substrate and Modified Membranes Characterization

The results of the contact angle measurements are presented in Table 1. Table 2 shows the values of the effective electric double layer capacity at the membrane/depleted solution interface, as well as the ohmic resistance of the membranes found from the analysis of the high-frequency arc of the electrochemical impedance spectrum. 

As expected, perfluorosulfonic acid polymer casting onto the surface of the heterogeneous substrate MK-40 membrane leads to an increase in hydrophobicity of the surface that results in the higher value of contact angle (sample MK-40_21_). This is attributed the less roughened surface of the modified membranes. Indeed, according to Wenzel [59], the effect of the surface roughness is to magnify its wetting properties: if the surface is hydrophilic, it will be more hydrophilic, the rougher the surface. Applying the PFSA layer results in flattening the surface of the modified membrane compared to the substrate membrane, which leads to lower hydrophilicity of the modified membrane surface and, consequently, the higher contact angle.

Adding the titanium dioxide nanoparticles into the modifying film (samples MK-40_21+3%_, MK-40_21+6%_, MK-40_33+3%_, and MK-40_33+6%_) results in increasing hydrophilicity of the membrane surface with increasing TiO_2_ mass fraction due to hydrophilic properties of the particles [60] and as the result of the increase in its roughness [61].

**Figure 2:**
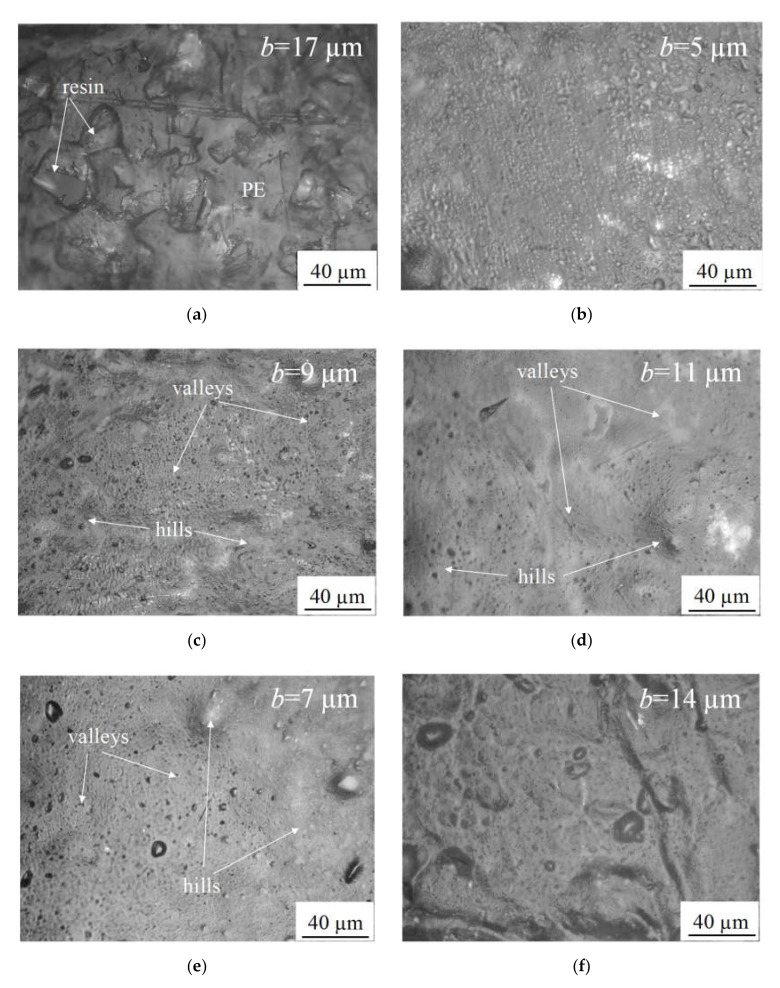
Optical images of the studied membranes: MK-40 (**a**), MK-40_21_ (**b**), MK-40_21+3%_ (**c**), MK-40_21+6%_ (**d**), MK-40_33+3%_ (**e**), MK-40_33+6%_ (**f**). Parameter *b* characterizes the roughness of the surface and shows the difference between the highest and lowest areas of the membrane relief.

Figure 2 shows that applying the PFSA layer results in flattening the surface of the modified membrane compared to the substrate membrane. Adding the titanium dioxide nanoparticles into the modifying film results in decreasing the flattening effect. The greater the fraction of TiO_2_, the lesser the effect of the modifying film. However, all the modified membranes have the less roughened surface compared to the substrate membrane.

The application of a modifying film practically does not change the ohmic resistance of the studied membranes, but noticeably affects the effective electric capacitance of their interphase boundary (Table 2). This capacity increases slightly after depositing the PFSA polymer film (sample MK-40_21_) and increases noticeably after inserting TiO_2_ particles into this film (samples MK-40_21+3%_ and MK-40_21+6%_). Note that this increase is observed if the thickness of the modifying film remains almost constant. In the case where the thickness of the PFSA polymer film increases by 1.5 times (samples MK-40_33+3%_ and MK-40_33+6%_), the addition of TiO_2_ nanoparticles to it practically does not lead to changes in the effective capacitance of the interphase boundary. It is known from studies in the field of electrode kinetics that the capacitance of the interphase boundary increases with increasing electric charge of the dense part of the electric double layer [50,62]. Apparently, coating the entire surface of the MK-40_21_ sample with an ion-conducting film leads to an increase in its electric charge in comparison with the substrate membrane (MK-40 sample), about 80% of which is occupied by inert polyethylene [9,28,46]. The introduction of 3 wt% particles into this film (sample MK-40_21+3%_) increases the charge of the membrane/solution interface. A further increase in the percentage of nanoparticles in the modifying film leads to an increase in the roughness of the modifying film/depleted solution interface, as well as a reorientation of the side chains of PFSA polymer with sulfone fixed groups towards charged nanoparticles. The result of this reorientation is “repulsion” to the surface of the main chains of the modifying material that do not have an electric charge. Similar phenomena are discussed in the works [63,64]. Apparently, in the case of using thicker films (samples MK-40_33+3%_ and MK-40_33+6%_), the reorientation of the side chains becomes crucial. Therefore, the introduction of nanoparticles does not lead to noticeable changes in the effective capacitance of the interphase boundary. To make sure our assumptions are correct, in the future we plan to conduct a direct measurement of the charge of the samples under study.

**Figure 3:**
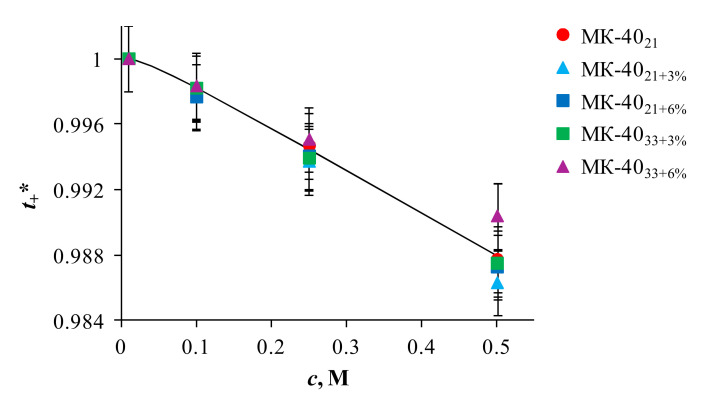
Concentration dependences of the conterion (Na^+^) transport number in the studied membranes.

An important parameter characterizing the membrane permselectivity is the counterion transport number, $t_{i}^{*}$. In the case of the studied cation-exchange membranes and sodium chloride solution, the value of $t_{+}^{*}$ shows the portion of the electric current transferred by the Na^+^ ion in conditions of the absence of diffusion. To evaluate the $t_{+}^{*}$ value, it is possible to use the following relationship, [65]:

(2) $$t_{-}^{*}=\frac{1}{2}-\sqrt{\frac{1}{4}-\frac{P^{*}F^{2}c}{2RT\kappa^{*}}},\;t_{+}^{*}=1-t_{-}^{*}$$

where $t_{-}^{*}$ is the transport number of coion; P^*^ and κ^*^ are the diffusion permeability and the electrical conductivity of the membrane, respectively; *c* is the concentration of the electrolyte solution; *F*, *R*, and *T* have their usual meanings. Using the experimentally determined values of electrical conductivity and diffusion permeability the counterion transport numbers in the studied membranes are calculated and presented in Figure 3 as function of NaCl solution concentration. It follows from this dependence that the modification of the samples practically does not change the counterion transport numbers in comparison with the substrate membrane. Values of $t_{i}^{*}$ remain high throughout the studied range of NaCl solution concentrations. The concentration dependencies of the conterion (Na^+^) transport number are approximated and extrapolated to the region of lower concentration (0.02 M), which corresponds to the measurements of the electrochemical characteristics (CVC, ChP, and impedance spectra). As a result, the values of the conterion transport number in the studied membranes at this concentration are virtually the same and equal to 1. 

### 3.2. Electroconvection and Water Splitting at the Surface of the Substrate Membrane and Modified Membranes

#### 3.2.1. Current-Voltage Curves

**Figure 4:**
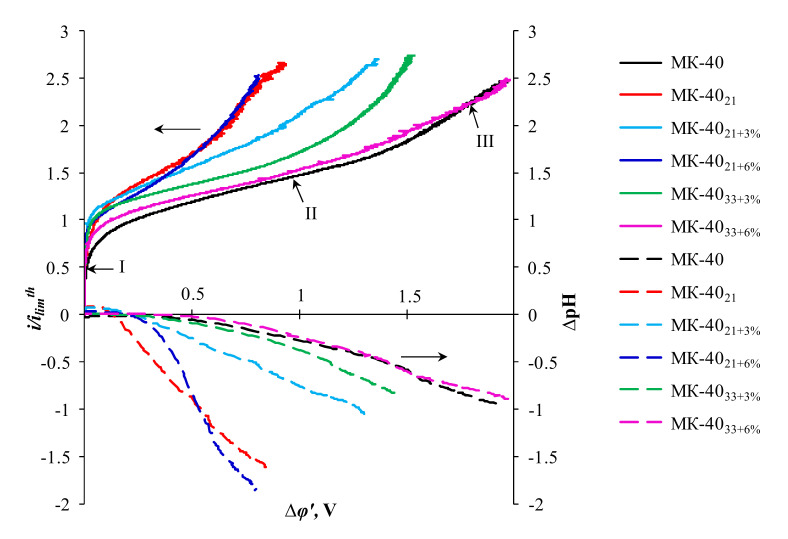
Current-voltage characteristics (solid lines) and the difference in pH between the outlet and inlet solution passing through the desalination compartment of the electrodialysis cell (dashed lines) of the pristine and modified membranes.

Figure 4 shows the ratio of the current density to its theoretical limiting value, $i/i_{\lim}^{\mathrm{th}}$ as well as the difference in the pH between the outlet (pH*_out_*) and inlet (pH*_in_*) solution passing through the desalination compartment (DC) of the electrodialysis cell, ΔpH (ΔpH = pH*_out_* − pH*_in_)*, as functions of reduced potential drop, ∆*φ’*. This is a potential drop between the Luggin’s capillaries, from which the ohmic component is subtracted. The exact definition of ∆*φ’* is given in the Supplementary Materials. The value $i_{\lim}^{\mathrm{th}}$ was calculated using the Leveque equation [66], which was derived in the framework of the convective-diffusion model under the assumption that the membrane has a smooth, homogeneous ion-conducting surface. In the conditions of our experiments, this calculation gives $i_{\lim}^{\mathrm{th}}$ = 2.01 mA cm^−2^ (see Supplementary Materials).

Three sections can be distinguished on the CVC [12,67]: an initial section (I), a slope plateau (II), and a section of a sharp growth of current density (III). It is believed [13,25,35], that the increase in the experimental current over the theoretical value $i_{\lim}^{\mathrm{th}}$ is determined by the EC, which develops according to the mechanism of electroosmosis of the first kind. The slope of the plateau characterizes the increase in the depleted diffusion layer conductivity as compared to that achieved at $i_{\lim}^{\mathrm{th}}$ due to the development of equilibrium EC [12,19]. The plateau length (determined by the potential drop, at which section II of the CVC turns into section III) corresponds to the threshold value of the potential drop at which a nonequilibrium EC occurs [12,19]. These characteristic parameters found by graphical treatment of the CVC (Figure 4) are summarized in Table 3.

Before discussing the obtained results, we recall that the modification of the membranes did not lead to any noticeable change in their selectivity (Figure 3), which could affect the values of the limiting currents.

**Figure 5:**
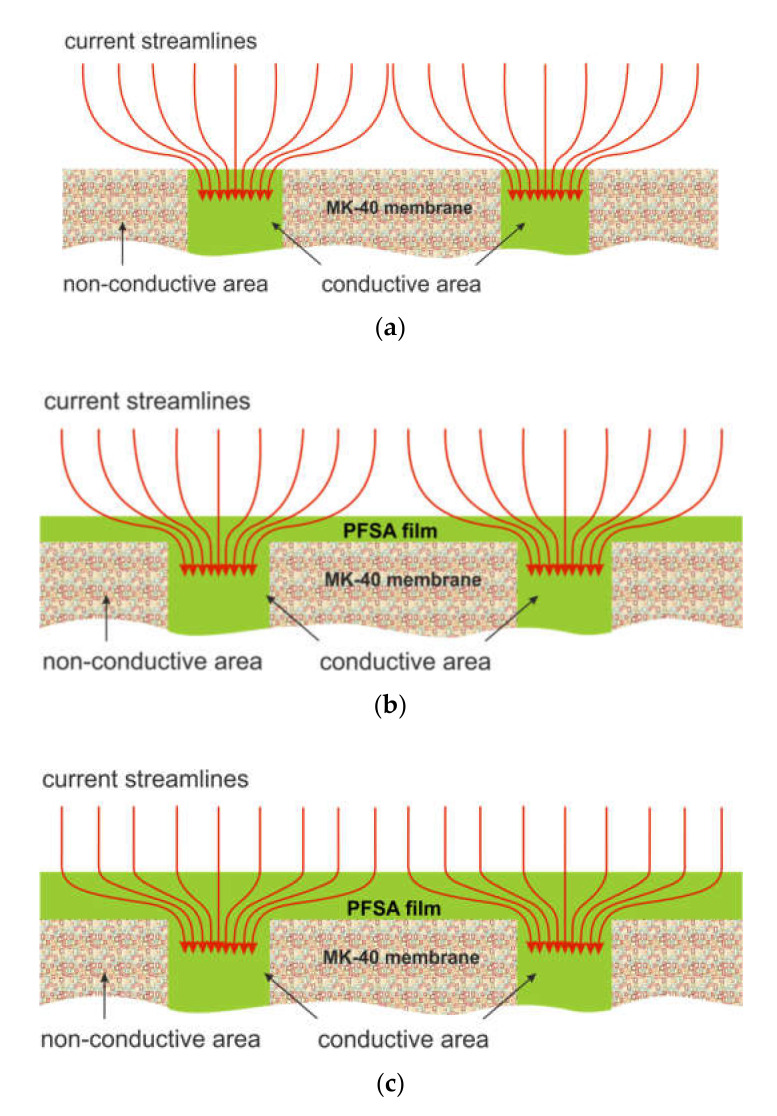
Schematic representation of streamlines near the surface of the MK-40 (**a**) membrane and that, modified by PFSA polymer film of various thicknesses: about 20 μm (**b**) and about 30 μm (**c**).

From these data it follows that in the case of the MK-40 substrate membrane the value $i_{\lim}^{\exp}/i_{\lim}^{\mathrm{th}}$ is less than unity. This result is expected, because only 20% of the surface of this membrane conducts electric current. The increase in current above the value $i_{\lim}^{\exp}/i_{\lim}^{\mathrm{th}}$ *=* 0.20 (which would correspond to the fraction of the conductive surface) is due to the tangential transport of salt from non-conductive regions to conductive ones, as well as due to electroconvection. The latter is stimulated by the “funnel effect” described by Rubinstein et al. [68], that is, curvature of streamlines and their crowding at inclusions of ion-exchange resin particles in polyethylene on the MK-40 surface [20] (Figure 5a). The value of the potential drop (plateau length), at which the development of a nonequilibrium EC begins, is close to the theoretically predicted one in the articles by Rubinstein and Zaltsman [12]. 

As in previous works [20,28], the deposition of the PFSA polymer film is accompanied by an increase in the $i_{\lim}^{\exp}/i_{\lim}^{\mathrm{th}}$ ratio, a decrease in the plateau length, and an increase in the conductivity of the depleted diffusion layer. This is facilitated by a decrease in surface hydrophilicity (Table 1), as well as by an increase in ion-conducting gates through which streamlines enter the ion-exchange membrane. Inserting 3 wt% TiO_2_ into the PFSA polymer film (sample MK-40_21+3%_) stimulates the development of all types of EC. Indeed, the $i_{\lim}^{\exp}/i_{\lim}^{\mathrm{th}}$ ratio, which is an indicator of the contribution of electroosmosis of the first kind to the increase in near-surface salt concentration, increases (Table 3). The plateau length, that is, the threshold potential drop at which a nonequilibrium EC occurs, is reduced in comparison with the MK-40 and MK-40_21_ samples. Apparently, this is facilitated by an increase in the electric charge while maintaining a rather high degree of hydrophobicity of the MK-40_21+3%_ sample surface (see Section 3.1). 

Note that even a slight increase in surface hydrophilicity while maintaining almost the same value of the effective capacitance of the interphase boundary of the modifying film/depleted solution (see Section 3.1) led to a decrease in the $i_{\lim}^{\exp}/i_{\lim}^{\mathrm{th}}$ value in the case of MK-40_21+6%_ sample as compared to MK-40_21+3%_. Nevertheless, in the case of MK-40_21+6%_ sample, the plateau length turned out to be minimal, and the plateau inclination turned out to be maximum among all the studied membranes. It can be assumed that the ratio of surface characteristics achieved in this sample turned out to be the most optimal for facilitating the development of nonequilibrium EC. Which of these characteristics became decisive for the stimulation of this type of EC is to be determined in subsequent experiments.

MK-40_33+3%_ and MK-40_33+6%_ samples, which were modified with a thicker PFSA polymer film, demonstrated a more modest ability to stimulate EC in comparison with not only MK-40_21+3%_ and MK-40_21+6%_ samples, but also with a sample that does not contain TiO_2_ particles. The reasons for such a behavior of the MK-40_33+3%_ and MK-40_33+6%_ samples were already discussed in Section 3.1. In addition, it can be assumed that at a thickness of the modifying film of about 20 μm (MK-40_21_, MK-40_21+3%_, MK-40_21+6%_ samples), the streamlines remain sufficiently curved to stimulate the increase in the tangential component of the electric force at the modifying film/depleted diffusion layer (Figure 5b). A 1.5-fold increase in the thickness of this film (MK-40_33+3%_ and MK-40_33+6%_ samples) apparently weakens the curvature of streamlines in the near-membrane layer of the solution, thereby reducing the tangential component of the electric force (Figure 5c).

#### 3.2.2. Chronopotentiometric Curves

Chronopotentiometry data provide additional details on the effect of modification on the degree of the membrane surface heterogeneity and on the mechanisms of EC development in the studied membrane systems.

**Figure 6:**
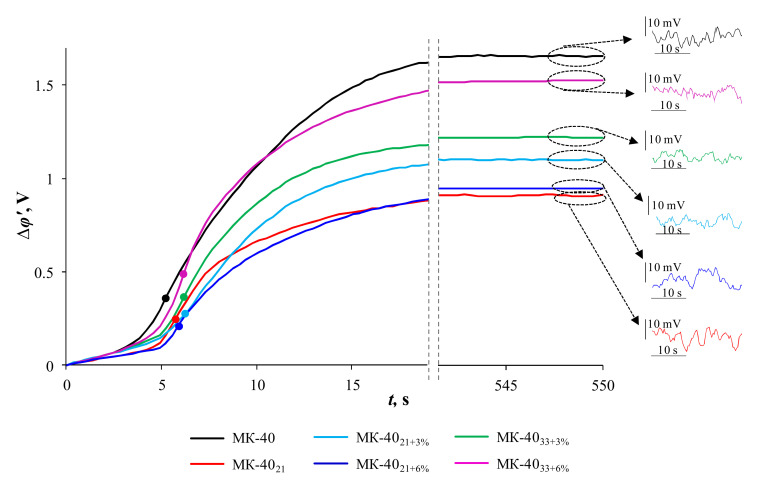
Chronopotentiometric curves of the studied membranes measured at $i/i_{\lim}^{\mathrm{th}}$= 2. The circles show the inflection points related to *τ_exp._*

Figure 6 shows the chronopotentiometric curves, ChPs, of the studied membranes obtained at $i/i_{\lim}^{\mathrm{th}}$ = 2. When plotting ChP, the reduced potential drop, ∆*φ’,* [69] is used (see the Supplementary Materials). Table 4 summarizes the transition times, *τ_exp_*, for various $i/i_{\lim}^{\mathrm{th}}$. They were determined by the inflection points on the initial sections of the ChPs (indicated by points in Figure 6). According to the classical Sand theory [70] and later studies [71], the inflection point on the ChP corresponds to the state of the system where the electrolyte concentration at the membrane surface decreases to minimum values, and the new mechanism of electric charge transfer begins to play a dominant role. These can be large electroconvection vortices that develop according to the mechanism of nonequilibrium electroconvection and deliver a more concentrated electrolyte solution to the membrane surface, and/or the water splitting, which is a source of new charge carriers (H^+^/OH^–^) ions [72]. A number of recent studies have shown that equilibrium electroconvection, which develops according to the mechanism of electroosmosis of the first kind, contributes to an increase in the transition times [25,35]. Small EC vortices formed by this mechanism cannot completely obviate the diffusion limitations of electrolyte delivery, which increase with increasing time from the moment the current is turned on. However, the delivery of a slightly more concentrated solution from the depleted diffusion layer shifts the moment of reaching the minimum surface concentration to greater times.

From the obtained data (Figure 6, Table 4) it follows that any modification of the MK-40 substrate membrane contributes to an increase in the transition times. The greatest differences in the behavior of the modified samples are observed in the current range 1.25 < $i/i_{\lim}^{\mathrm{th}}$ < 2.5. Moreover, the best ability to stimulate equilibrium EC, which is expressed in the prolongation of transition times, demonstrate MK-40_21+3%_ and MK-40_33+3%_ samples. These results are consistent with voltammetry data (Section 3.2.1). Apparently, at too high currents, the values of the transition times are reached so quickly that the equilibrium EC does not have time to fully develop. 

As already mentioned in Section 1, in the case of a nonequilibrium EC, large vortices form clusters. Under conditions of forced convection of the solution, these clusters move along the surface of the membranes under the measuring capillaries, which is expressed in the oscillations of the recorded potential drop. We registered such oscillations for all studied membranes (Figure 6). The largest amplitude of oscillations, which indicates the development of larger EC cluster structures, is demonstrated by MK-40_21_ and MK-40_21+6%_ samples. This result also confirms the conclusions made by us after analyzing the CVCs.

It should be noted that the chronopotentiometry data allow one to assess the degree of membrane surface electrical heterogeneity: the higher this heterogeneity, the faster the potential drop increases in the first few seconds after turning on the current [71]. From the obtained data (Figure 6), it follows that the surface of the MK-40_21_ and MK-40_21+6%_ samples is less electrically heterogeneous in comparison with other studied membranes. This experimental fact allows one to conclude that the high ability of the MK-40_21+6%_ sample to stimulate EC is mainly caused by an increase in the electric charge of the modifying film/solution interface rather than by an increase in the surface roughness after introducing 6 %wt TiO_2_ into the PFSA polymer film.

#### 3.2.3. Impedance Spectra under Direct Current Condition and the pH Difference at the Outlet and Inlet of the Desalination Channel of the Electrodialysis Cell

**Figure 7:**
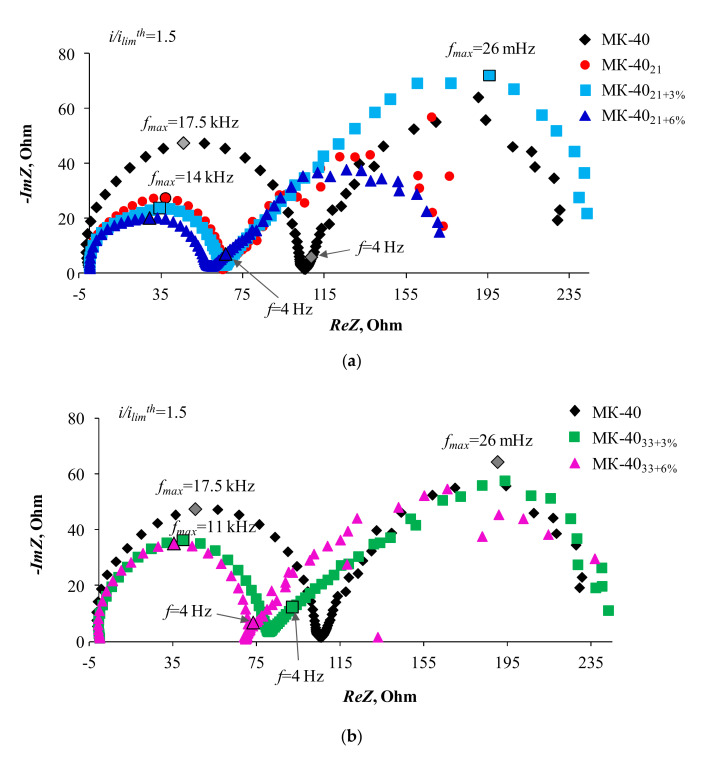
Electrochemical impedance spectra of the MK-40 as well as MK-40_21_, MK-40_21+3%_, MK-40_21+6%_ (**a**) and MK-40_33+3%_, MK-40_33+6%_ (**b**) samples at $i/i_{\lim}^{\mathrm{th}}$ = 1.5.

**Figure 8:**
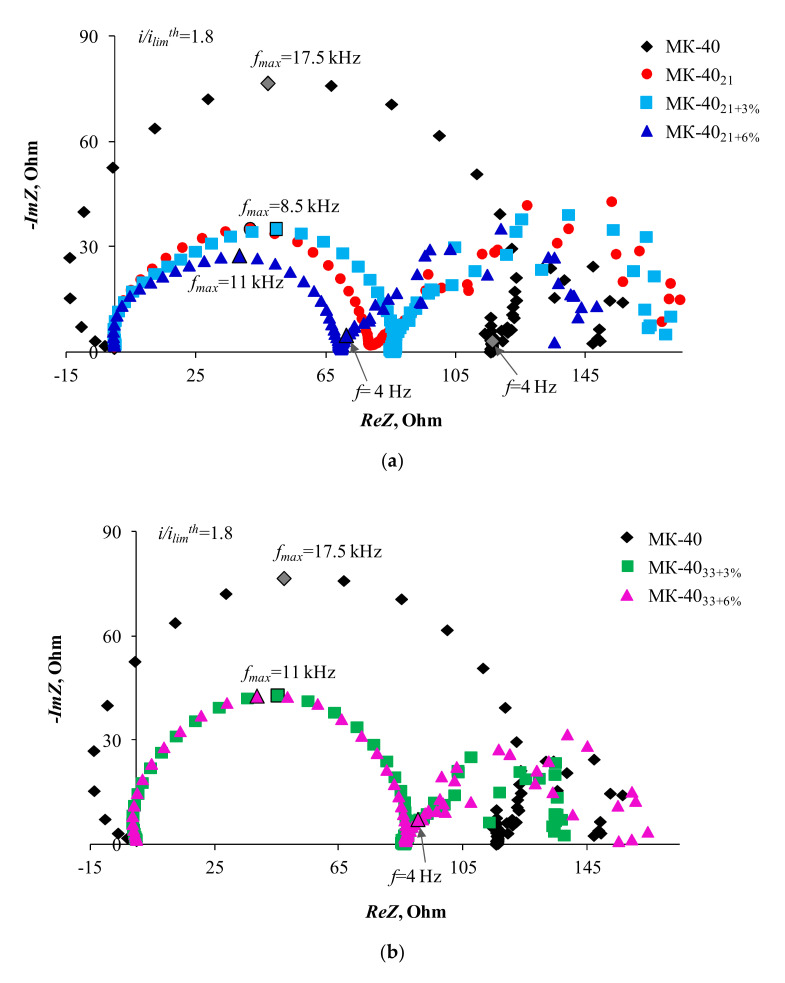
Electrochemical impedance spectra of the MK-40 as well as MK-40_21_, MK-40_21+3%_, MK-40_21+6%_ (**a**) and MK-40_33+3%_, MK-40_33+6%_ (**b**) samples at $i/i_{\lim}^{\mathrm{th}}$ = 1.8.

Figure 7 and Figure 8 show the electrochemical impedance spectra obtained for the studied membranes at 1.5 and 1.8 $i/i_{\lim}^{\mathrm{th}}$. All these spectra consist of two arcs. The first high-frequency arc is characterized by frequencies at the point of maximum along the ordinate axis from 8.5 to 17.5 kHz. Similar characteristic frequencies for this arc (5–11 kHz) are demonstrated by the homogeneous cation-exchange membrane CMX (Astom, Tokyo, Japan), studied under similar conditions [73].

The length of the high-frequency arc chord corresponds to the resistance of the membrane system, R_I_ (in our case, mainly a depleted diffusion layer), which depends on the degree of polarization caused by direct current [48,49]. From the obtained data, it follows that the maximum value of such resistance at a given direct current is achieved in the case of a substrate membrane MK-40. Any modification of its surface leads to a decrease in R_I_. The values decrease in the same series in which the ability of membranes to stimulate EC increases: MK-40 > MK-40_33+6%_ ≥ MK-40_33+3%_ > MK-40_21+3%_ > MK-40_21+6%_ ≈ MK-40_21_. The appearance of the Warburg impedance arc is caused by the time delay of changes in the electrolyte concentration in the quasi-electroneutral layer of the solution adjacent to the membrane in response to the alternating current bias [49,74,75]. According to the classical assumptions [49,75], the reason for this delay (and the delay caused by it in changes in the potential drop) is the finite rate of the electrolyte diffusion. According to modern concepts, this rate can change in the presence of electroconvective vortices at the membrane surface. Their appearance is accompanied by distortion in the regular shape of the arc. The more intensive electroconvective mixing, the stronger the spread of points in this region of the spectrum. The characteristic value of the frequency at the maximum point of the Warburg arc is 10 mHz [35,73]. Our results confirm that the second arc corresponds to Warburg impedance. Moreover, the effect of electroconvection on this arc (expressed in an increasing spread of points), increases with increasing density of the overlimiting current. This effect is characteristic for all the studied samples. Apparently, the electroconvective mixing provides a sufficiently high salt concentration at the membrane/solution interface. As a result, even at sufficiently high current densities (Figure 7 and Figure 8), the Gerischer arc, which appears in the case of a homogeneous CMX membrane with the same sulfonic fixed groups at $i/i_{\lim}^{\mathrm{th}}$ = 1.6, cannot be identified on the impedance spectra. As mentioned earlier, the Gerischer impedance [54] corresponds to the resistance caused by a chemical reaction (in our case, water splitting) at the membrane surface/depleted diffusion layer interface. In the case of the CMX membrane, the characteristic frequencies corresponding to the maximum points of this arc have values of about 4 Hz. In the considered systems, these values are situated in the region of the spectrum where the second arc is bordering the high-frequency arc. Note that this region is characterized by the absence of spread of points and does not represent a straight line passing at a 45° angle to the Re*Z* axis (such characteristics should have the initial part of the finite-length Warburg impedance [50]). Apparently, the deviation of this part of the spectrum from the classical form is caused by the influence of water splitting. An indirect evidence of this reaction, at least at high current densities, is the fact that the Re*Z* takes a negative value, recorded in the case of MK-40 ($i/i_{\lim}^{\mathrm{th}}$= 1.8) in the frequency range 35–500 kHz. A similar shape of spectra was observed in the case of water splitting for many membrane systems [35,73]. However, the rate of this reaction in studied systems is so small that the Gerischer arch does not appear.

Nevertheless, this water splitting at the cation-exchange membrane/solution interface, affects the pH difference at the outlet and inlet of the desalination channel, which is formed by the studied cation-exchange membranes and the MA-41 anion exchange membrane. This follows from Figure 4 that shows the difference in the pH between the outlet and the inlet solution passing through the desalination compartment of the electrodialysis cell, ΔpH, as functions of reduced potential drop, ∆*φ’*. The MA-41 membrane contains a certain amount of secondary and tertiary amines, whose catalytic activity with respect to the water dissociation reaction is quite high [76,77]. The protons generated on the surface of this membrane acidify the desalinated solution. As a result, ΔpH of the solution is less than zero. In the case where the water splitting also occurs on the surface of the cation-exchange membrane, hydroxyl ions enter the solution. As a result, ΔpH of the solution should tend to zero or even positive values. The closer the ΔpH of the solution to zero, the more intensive the water splitting on the surface of the cation-exchange membrane. Indeed, in intensive current modes, the water splitting rate on the MK-40_33+6%_ surface is close to that observed on the MK-40 substrate membrane. The lowest water splitting rate occurs in the case of MK-40_21_ and MK-40_21+6%_ samples. As already mentioned, the cause of the decrease in water splitting is electroconvection, which shifts intensive water splitting to the range of higher potential drops.

## 4. Conclusions

The surface of the MK-40 heterogeneous cation-exchange membrane was modified with a Nafion-type PFSA polymer film containing TiO_2_ nanoparticles.

It was shown that such modification does not affect the selectivity of the obtained samples. All of them, like the MK-40 substrate membrane, are characterized by counterion transport numbers (Na^+^) close to 1. Introduction of TiO_2_ nanoparticles (from 3 to 6 wt%) into the modifying film leads to an increase in surface charge while maintaining a sufficiently low degree of its hydrophilicity.

These changes in surface characteristics result in an increase of the experimental limiting current density on 9–39% due to the stimulation of electroconvection, developing by the mechanism of electroosmosis of the first kind. The greatest increase in current as compared to the substrate membrane can be obtained at a particle concentration of 3 wt%. A sample containing 6 wt% provides greater mass transfer in overlimiting current modes due to the development of nonequilibrium electroconvection. Electroconvective mixing of the solution leads to an increase in the near-surface salt concentration as compared to the substrate membrane. As a result, water splitting on the surface of modified samples exerts an extremely insignificant effect on their mass transfer characteristics in intensive current modes.

The above results are achieved with a thickness of the modifying film equal to 20 μm. A 1.5-fold increase in the thickness of this film reduces the positive effect of the introduction of TiO_2_ nanoparticles due to (1) partial shielding of the nanoparticles on the surface of the modified membrane; (2) a decrease in the tangential component of the electric force, which affects the development of electroconvection.

The tested relatively simple method of surface modification of commercial membranes can be used to increase mass transfer and increase their resistivity to sedimentation and fouling in the processes of electrodialysis processing of natural water and liquid media of the food industry.

## Figures and Tables

**Table 1:** Studied membranes and some characteristics.

Sample	Modifying Film Thickness^1^, μm	Mass Fraction TiO_2_ in the Film, wt%	Contact Angles, *θ*, Degrees (Swollen Membrane)
MK-40	–	–	55 ± 2
MK-40_21_	21 ± 2	–	64 ± 3
MK-40_21+3%_	21 ± 2	3	61 ± 3
MK-40_21+6%_	21 ± 2	6	57 ± 2
MK-40_33+3%_	33 ± 2	3	60 ± 3
MK-40_33+6%_	33 ± 2	6	56 ± 2
^1^ in swollen state			

All membrane samples underwent standard salt pretreatment [47] before the measurements.

**Table 2:** Characteristic points of the high-frequency region of the studied membranes impedance spectra and the effective capacities of the electric double layer and the ohmic resistances of the membranes found from these points.

Sample	*f*_max_, Hz	−Im*Z*_max_, Ohm	*R*^Ω^, Ohm	*C*, μF
MK-40	13,318	22.2	55 ± 2	0.5 ± 0.2
MK-40_21_	10,809	21.7	54 ± 2	0.7 ± 0.2
MK-40_21+3%_	8506	19.0	50 ± 2	1.0 ± 0.2
MK-40_21+6%_	8506	20.4	49 ± 2	0.9 ± 0.2
MK-40_33+3%_	10,809	20.2	51 ± 2	0.7 ± 0.2
MK-40_33+6%_	10,809	21.8	52 ± 2	0.7 ± 0.2

**Table 3:** Some parameters of current-voltage curves, current-voltage characteristics (CVCs).

Sample	$i_{\lim}^{\exp},\;\mathrm{mA}\;\mathrm{cm}{–}^{2}$	$i_{\lim}^{\exp}$ $/i_{\lim}^{\mathrm{th}}$	Plateau Length, V	Plateau Slope, mS cm^–2^
MK-40	1.57	0.78	1.43	0.61
MK-40_21_	1.81	0.90	0.60	1.58
MK-40_21+3%_	2.19	1.09	0.85	1.08
MK-40_21+6%_	1.97	0.98	0.50	1.33
MK-40_33+3%_	2.01	1.00	1.03	0.78
MK-40_33+6%_	1.71	0.85	1.25	0.62

**Table 4:** Values of the experimental transition time, *τ*_exp_, at various $i/i_{\lim}^{\mathrm{th}}$ for the studied membranes.

$i/i_{\lim}^{\mathrm{th}}$	MK-40	MK-40_21_	MK-40_21+3%_	MK-40_21+6%_	MK-40_33+3%_	MK-40_33+6%_
1.25	15.2 ± 0.2	18.6 ± 0.2	18.4 ± 0.2	17.5 ± 0.2	17.7 ± 0.2	17.6 ± 0.2
1.5	9.2 ± 0.2	10.5 ± 0.2	12.1 ± 0.2	9.9 ± 0.2	11.8 ± 0.2	11.7 ± 0.2
2.0	5.2 ± 0.2	5.7 ± 0.2	6.2 ± 0.2	5.9 ± 0.2	6.1 ± 0.2	6.1 ± 0.2
2.5	2.9 ± 0.2	3.6 ± 0.2	3.8 ± 0.2	3.7 ± 0.2	3.8 ± 0.2	3.8 ± 0.2

## Supplementary Materials

Supplementary materials can be found at https://www.mdpi.com/2077-0375/10/6/125/s1. Figure S1: Principal scheme of the experimental setup: electrodialysis cell (1) consisting of one desalination compartment (DC), one concentration compartment (CC) and two electrode compartments; tanks with solutions (2, 3); Luggin’s capillaries (4), connected with silver chloride electrodes (5); electrochemical complex Autolab PGSTAT-100 (6); flow pass cell with pH combination electrode (7), connected to pH-meter Expert 001 (8). Figure S2: The experimental setup for contact angles measurements: dispenser of the test liquid (1); closed optically transparent box (2); camera with sufficient magnification (3); drop of the test liquid (4); test sample (5); filter paper (6); layer of the test liquid (7); sample stand (8).
