# Supplementary material for: The Development of Electroconvection at the Surface of a Heterogeneous Cation-Exchange Membrane Modified with Perfluorosulfonic Acid Polymer Film Containing Titanium Oxide
Source: Membranes (Basel). 2020 Jun 17;10(6):125. doi: 10.3390/membranes10060125 (PMC7344879; doi:10.3390/membranes10060125)
Supplement: Supplementary file 1 [file membranes-10-00125-s001.pdf]

membranes

Article

Supplementary Materials: The Development of Electroconvection at the Surface of a Heterogeneous Cation-Exchange Membrane Modified with Perfluorosulfonic Acid Polymer Film Containing Titanium Oxide

Violetta Gil *, Mikhail Porozhnyy, Olesya Rybalkina, Dmitrii Butylskii and Natalia Pismenskaya

Department of Physical Chemistry, Kuban State University, 149 Stavropolskaya st., 350040 Krasnodar, Russia; porozhnyj@mail.ru (M.P.); olesia93rus@mail.ru (O.R.); dmitrybutylsky@mail.ru (D.B.); n_pismen@mail.ru (N.P.)

* Correspondence:Correspondence: violetta_gil@mail.ru

Received: 12 May 2020; Accepted: 15 June 2020; Published: date

The Experimental Setup for Electrochemical Characteristics Measurements

The electrochemical characteristics, the current-voltage curves, chronopotentiograms and impedance spectra of the studied membranes are obtained using the experimental setup shown in Fig. S1. The setup includes a laboratory four-compartment flow electrodialysis cell (1), described in details in [45]. The compartments of the cell are formed by the studied cation-exchange membrane (C*) and auxiliary anion-exchange MA-41 (A) and cation-exchange MK-40 (C) membranes. The auxiliary membranes protect the studied membrane from the products of electrode reactions. The distance between the neighboring membranes is 6.6 mm, the polarized membrane area is 2 × 2 cm^2. The desalination compartment (DC) is fed with a NaCl solution from one tank (2), the concentration compartment (CC) and electrode compartments are supplied from the other tank (3). The average linear flow velocity of the solutions in all compartments is equal to 0.4 cm/s, the initial concentration of the solutions is equal to 0.02 M.

A potential drop across the studied membrane is determined using two Luggin's capillaries (4), connected with silver chloride electrodes (5). Current is supplied to the cell and the potential drop was measured using an electrochemical complex Autolab PGSTAT-100 (EcoChemi, Utrecht, The Netherlands) (6).

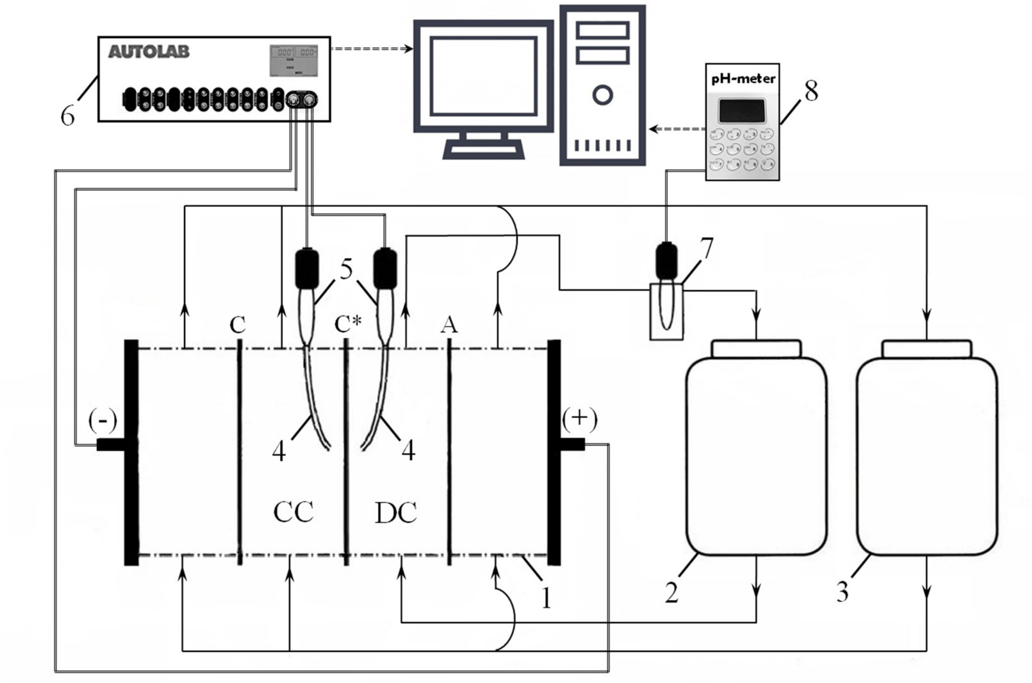

Figure S1. Principal scheme of the experimental setup: electrodialysis cell (1) consisting of one desalination compartment (DC), one concentration compartment (CC) and two electrode compartments; tanks with solutions (2, 3); Luggin's capillaries (4), connected with silver chloride electrodes (5); electrochemical complex Autolab PGSTAT-100 (6); flow pass cell with pH combination electrode (7), connected to pH-meter Expert 001 (8).

During the voltammetry and chronopotentiometry measurements the pH of the desalted solution at the DC outlet (pH_{out}) is recorded using a flow pass cell with a pH combination electrode (7) and pH-meter Expert 001 (Econix-Expert, Ltd., Moscow, Russia) (8). The pH of the solution at the inlet of DC (pH_{in}) is controlled in tank (2).

The electrodialysis cell (1) design (in particular, the special input and output devices of the solution) provide a laminar flow of the solution in the intermembrane space [45]. The laminarity of the hydraulic regime was proved by using CFD simulations and by comparing the experimental limiting current density with the value calculated using the Lévêque equation, which was deduced under the assumption of laminar flow in the intermembrane space.

The theoretical limiting current density, $i_{\lim}^{\mathrm{th}}$, which is achieved in the absence of coupled effects of concentration polarization (such as electroconvection, gravitational convection, and water splitting) can be calculated by the Lévêque equation [66]:

$$i_{\lim}^{\mathrm{th}} = \frac{FDz_1C_1}{h\left(T_1 - t_1\right)}\left[\left(1.47\frac{h^2V}{LD}\right)^{1/3} - 0.2\right] \quad (1)$$

where F is the Faraday constant; D is the electrolyte diffusion coefficient; z_1 and C_1 are the charge and the input molar concentration of the counterion; T_1 and t_1 are the effective transport number of the counterion (Cl^- in the considered cases) in the membrane and its transport number in solution; V is the average linear flow velocity; L is the length of the desalination path; h is the intermembrane distance.

The parameters used in the experiments (at 25 °C) are as follows: $D = 1.61 \times 10^{-9}$ m²/s, T_1 is assumed to be 1, t_1 is 0.396, V is 0.4 cm·s^{-1}, h is 6.6 mm, and L is 2 cm. The value calculated from Eq. (1) for these experimental conditions is 2.01 mA·cm^{-2}.

The Experimental Setup for Contact Angles Measurements

The contact angles on the membrane surface, in the sodium form, were measured by the sessile drop technique. This technique [20] differs from the traditional ones by that the membrane is in swollen state, pre-equilibrated with a solution (0.02 M NaCl). The scheme of the experimental setup is shown in Fig. S2. A drop of distilled water of approximately 7 µL in volume was applied from a height of 0.7 cm on the membrane surface. The membrane was removed from the equilibration solution just before the measurements. It is placed in a closed optically transparent box on a filter paper, which is in contact with the test liquid. The residual solution film on the membrane side faced to the liquid dozer is quickly removed. The images obtained with a digital video camera are processed using computer program ImageJ to improve the contrast of the contours of the drop. 20 s after the application of a drop, the inflow wetting angle on the membrane surface is found using the tangent method. The experiment is repeated no less than 10 times. The drop is placed onto various parts of the membrane surface; then the average value of the contact angle and the inaccuracy is calculated. The standard deviation of determining the angle is 2–3°. This method allows keeping the membrane in conditions close to thermodynamic equilibrium with a solution. Moreover, these conditions are similar to the real state of membrane in electrodialysis.

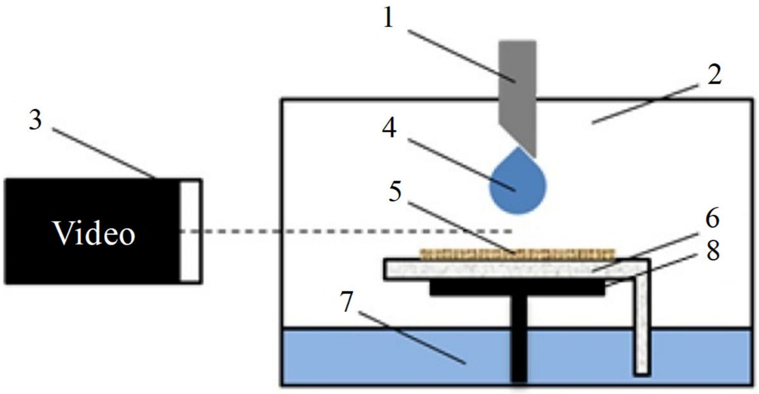

Figure S2. The experimental setup for contact angles measurements: dispenser of the test liquid (1); closed optically transparent box (2); camera with sufficient magnification (3); drop of the test liquid (4); test sample (5); filter paper (6); layer of the test liquid (7); sample stand (8).

Processing of Experimental Data

The experimental current-voltage curves are plotted in the coordinates of the ratio of the current density to its theoretical limiting value $i / i_{\lim}^{\text{th}}$ versus the reduced potential drop $\Delta\varphi'$. In this case $\Delta\varphi'$ is related to the measured potential drop as

$$\Delta\varphi' = \Delta\varphi - iR_{i=0} \qquad (2)$$

where $\Delta\varphi'$ is the measured value of potential drop at a current density i and $R_{i=0} = \left(\partial\Delta\varphi / \partial i\right)_{i\to 0}$ is the resistance of the membrane system at $i \to 0$.

To compare chronopotentiograms of different membrane systems, it is convenient to use the reduced potential drop $\Delta\varphi'$ [69] instead of the total potential drop, $\Delta\varphi$:

$$\Delta\varphi' = \Delta\varphi - \Delta\varphi_{ohm} \qquad (3)$$

where the ohmic potential drop, $\Delta\varphi_{ohm}$, of the unpolarized membrane system is found by the extrapolation in the $\Delta\varphi - \sqrt{t}$ coordinates to zero time (the time of the current switch-on).

© 2020 by the authors. Submitted for possible open access publication under the terms and conditions of the Creative Commons Attribution (CC BY) license

(http://creativecommons.org/licenses/by/4.0/).
